# Socioeconomic Inequalities in the Prevalence of Pit and Fissure Sealant Use Among Mexican Adolescents Aged 12 and 15: Implications for Public Policy

**DOI:** 10.7759/cureus.83980

**Published:** 2025-05-12

**Authors:** América P Pontigo-Loyola, María de L Márquez-Corona, Martha Mendoza-Rodríguez, Salvador E Lucas-Rincón, Juan J Villalobos-Rodelo, Alejandro J Casanova-Rosado, Mauricio Escoffié-Ramírez, Rosalina Islas-Zarazúa, Carlo E Medina-Solís, Sonia Márquez-Rodríguez

**Affiliations:** 1 Academic Area of Dentistry of Health Sciences Institute, Autonomous University of Hidalgo State, Pachuca, MEX; 2 School of Dentistry, Autonomous University of Sinaloa, Culiacán, MEX; 3 School of Dentistry, Autonomous University of Campeche, Campeche, MEX; 4 School of Dentistry, Autonomous University of Yucatan, Mérida, MEX; 5 Advanced Studies and Research Center in Dentistry "Dr. Keisaburo Miyata" School of Dentistry, Autonomous University of the State of Mexico, Toluca, MEX

**Keywords:** mexico, oral health, pit and fissure sealants, prevention, tooth decay

## Abstract

Background

Oral diseases are a public health problem due to their high global prevalence and incidence and their significant health and economic burden. Despite being preventable, dental caries remains the most common chronic disease affecting children and adolescents globally. Caries prevention relies on multifaceted strategies, and pit and fissure sealants (PFS) are a cost-effective intervention, physically preventing biofilm accumulation on occlusal surfaces and thereby reducing caries. The objective of this study was to determine the existence of socioeconomic inequalities in the prevalence of pit and fissure sealant (PFS) use in Mexican adolescents aged 12 and 15 years.

Materials and methods

An observational, cross-sectional study was conducted in 1,538 Mexican adolescents aged 12 and 15 years. The presence of pit and fissure sealants was determined through a clinical examination and served as the dependent variable. This was dichotomized as follows: 0 = no presence of PFS and 1 = with at least one PFS. A questionnaire was administered to determine a series of independent sociodemographic variables and socioeconomic position (SEP) indicators. Statistical analysis was performed using Fisher's exact test, the chi-square test, and a non-parametric test for trend in Stata 14.0 (StataCorp LLC, College Station, TX).

Results

By age group, the highest percentage was found to be 15 years old (55.3%, n=850). Regarding sex, 50.1% (n=770) were boys. The prevalence of having any tooth with PFS was 1.3% (n=20). An association was observed between the prevalence of PFS and the SEP variables of education (p=0.047) and occupation (p=0.017). It was also observed that as the socioeconomic status (SES) tertiles increased (from the poorest to the richest), the prevalence of PFS also increased (p<0.05). Similarly, the prevalence of PFS was higher among those who did not always live in the community where they currently resided (2.3%, n=10 versus 0.9%, n=10; p=0.035) and in those who regularly visited the dentist (3.9%, n=9 versus 0.8%, n=11; p=0.001).

Conclusions

A very low prevalence of PFS use (1.3%, n=20) was observed in this sample of Mexican adolescents. The results suggest the existence of socioeconomic inequalities in the use of PFS and a low level of dental prevention behavior. The data show a clear trend where the use of PFS is much higher among teenagers from wealthier backgrounds compared to those from poorer backgrounds, and this pattern is seen in both education and job-related measures of socioeconomic status. It is necessary to implement PFS placement programs if oral health is to be improved.

## Introduction

Oral diseases are a public health problem due to their high global prevalence and incidence and their significant health and economic burden [1-3]. As with most health conditions, the greatest burden of oral diseases falls on economically disadvantaged and socially marginalized populations. Moreover, their severe impact, in terms of pain and suffering, functional impairment, and reduced quality of life, must be acknowledged. Conventional treatment for oral disorders is often prohibitively expensive in most low- and middle-income countries (LMICs) [4]. This situation has persisted for decades: according to the 2021 Global Burden of Disease report, the most prevalent oral diseases (untreated caries, severe periodontitis, edentulism, and oral cancer) affected 3.69 billion people worldwide, with a standardized prevalence of 45.9%. The most common conditions were untreated caries in permanent teeth (27.5%) and severe periodontitis (12.5%). In contrast, edentulism, periodontitis, and oral cancer accounted for the highest disease burden in disability-adjusted life years (DALYs) [5].

Despite being preventable, dental caries remains the most common chronic disease affecting children and adolescents globally. Caries not only compromises dental function and aesthetics but is also associated with severe pain, infections, poor academic performance, and frequent school absenteeism [1,6]. In childhood and adolescence, caries affecting the first permanent molars exacerbates the problem, as early loss of these teeth can lead to malocclusion, potentially necessitating complex and specialized dental procedures [7-9]. In Mexico, dental caries persists as a public health issue, with prevalence rates among children and adolescents ranking among the highest worldwide. Estimates indicate that 70%-85% of 12-year-olds exhibit caries in their permanent dentition, while 50% of six-year-olds have caries in their primary teeth [10]. Nationwide studies further report that caries affects approximately half of students aged 5-16 [11,12].

Caries prevention relies on multifaceted strategies, including oral health education (e.g., frequent toothbrushing, regular dental visits, and reduced refined sugar intake), fluoride use (e.g., toothpaste, mouth rinses, and professional applications), and community-based interventions, notably pit and fissure sealants (PFS), which create a physical barrier against bacteria, acids, and food debris [13-18]. PFS are a cost-effective intervention, physically preventing biofilm accumulation on occlusal surfaces and thereby reducing caries [19-23]. Their application to permanent molars in children and adolescents is widely recommended: randomized controlled trials (RCTs) with 2-3 years of follow-up demonstrate a 76% reduction in occlusal caries incidence. Comparative RCTs further show PFS to be 73% more effective than fluoride varnish in preventing occlusal caries over the same period [21]. However, in LMICs such as Mexico, PFS implementation faces challenges tied to inequitable access to preventive services and the absence of targeted public policies [24].

PFS prevalence varies significantly across countries, reflecting socioeconomic disparities. In high-income nations, studies report rates of 46.6%-33.8% [25], 43% [26], or 20% [19] among children aged 6-14 in the United States, 34.8% and 32.2% in 12- and 15-year-olds in Spain [27], and 9% in Saudi adolescents aged 12-14 [28]. In contrast, LMICs report far lower rates: 2.21% in São Tomé [29], 3.77% in Tibet [30], and 4.2% in Puerto Rico [31], with stark disparities by income, ethnicity, and education. In Mexico, a national study of 12-year-olds found that PFS use never exceeded 2.5% over 10 years [32]. Although there is strong proof that PFS works well [33-36], its use in Mexico is not well researched, showing that dental care mainly focuses on treating problems instead of preventing them.

Globally, oral health inequalities persist among children from minority groups, lower socioeconomic backgrounds, and those with limited access to dental care, disparities intrinsically linked to parental socioeconomic position (SEP) [6]. SEP encompasses social and economic dimensions (e.g., resources, prestige, and power), not just income. An individual's position in the social hierarchy is shaped by education, income, and occupation, and is further influenced by cultural norms and historical policies. SEP is critical for understanding how socioeconomic inequities impact health outcomes. Generally, poorer SEP correlates with worse health status, prompting research into explanatory mechanisms [37-40]. Access to care also mediates SEP-driven disparities, reflecting both systemic barriers (e.g., healthcare infrastructure) and individual-level interactions with local health institutions [37]. Literature confirms socioeconomic influences on PFS utilization. We hypothesize significant socioeconomic inequalities in PFS prevalence, with higher rates among wealthier strata and adolescents with regular dental access. Thus, this study aims to assess socioeconomic disparities in PFS use among Mexican adolescents aged 12 and 15.

## Materials and methods

Study design, population, and sample

**Figure 1:**
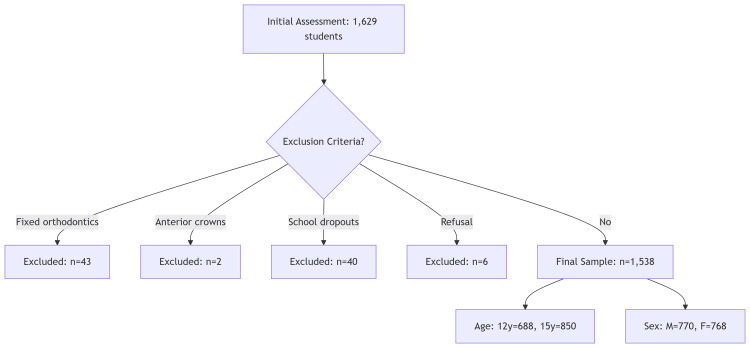
Flow diagram of participant selection process From an initial pool of 1,629 schoolchildren, 91 were excluded based on predefined criteria, yielding a final analytical sample of 1,538 adolescents (94.4% retention rate).

This observational cross-sectional study evaluated adolescents aged 12 and 15 years from public schools in three communities of Tula de Allende, Hidalgo, Mexico (Tula Centro, San Marcos, and El Llano), with an approximate duration of 10 months. The methodology has been previously published [24,41,42]. From an initial pool of 1,629 students across 25 schools, the inclusion criteria were as follows: (1) adolescents aged 12 and 15 years, (2) of either sex, (3) who attended primary or secondary school in Tula del Centro, San Marcos or El Llano, (4) who agreed to participate in the study and were available for the clinical examination, (5) and their parents signed the informed consent forms. The exclusions applied to 91 participants: 43 had fixed orthodontic appliances, two presented with full anterior crowns, 40 were school dropouts, and six refused examinations. The final analytical sample comprised 1,538 adolescents (Figure 1).

Variables and data collection

This work is part of a project that collected various oral health indicators. A pilot test was conducted to standardize criteria and verify the duration of procedures performed in the schoolchildren's clinical examinations. A probe and a size 5 mouth mirror were used for the clinical oral examination under daylight. The students were examined within the facilities of each educational institution. The oral examinations (for caries and fluorosis) were performed by two trained examiners who were standardized according to the criteria used (Kappa > 0.85). These results are not presented in this manuscript.

The primary outcome was pit and fissure sealant prevalence, categorized dichotomously as absent (0) or present (≥1 sealant).

Independent variables collected via maternal questionnaires included age (12 or 15 years), sex, current residence (Tula, El Llano, or San Marcos), residential stability history, dental visit frequency, health insurance status, maternal literacy, and socioeconomic position indicators. Socioeconomic position was derived through principal component analysis of parental education and occupation data, stratified into population tertiles with the highest tertile representing the most advantaged status.

Statistical analysis

Analyses included frequency distributions for all categorical variables. Bivariate testing employed Fisher's exact tests, chi-square tests, and non-parametric trend tests as appropriate. The low prevalence in key strata prevented meaningful multivariate analysis. All statistical procedures were conducted using Stata 14.0 (StataCorp LLC, College Station, TX).

Data visualization was implemented through graphical representations and tabular formats. Scatterplots with linear trendlines were generated using Microsoft Excel (Microsoft Corp., Redmond, WA), including display of regression equations and coefficient of determination (R²) values to quantify relationships.

Ethical considerations

The research strictly followed Mexican health research regulations and the ethical principles of the Declaration of Helsinki (2013). Before initiating the study, we secured written informed consent from all parents/guardians for the use of their children's anonymized clinical data. In addition, the consent of the adolescents was obtained. Ethical approval was obtained from the Ethics Committee of the Autonomous University of the State of Hidalgo, with subsequent validation for secondary data analysis by the Stomatology Research Network's Ethics Committee (CEIRIE-003.25). We implemented robust confidentiality measures, including data anonymization and secure storage systems, to ensure full compliance with contemporary data protection standards.

## Results

Sample characteristics and key findings

Table 1 presents the demographic and socioeconomic characteristics of the study population comprising 1,538 adolescents aged 12 and 15 years. The sample included slightly more 15-year-olds (55.3%, n=850) than 12-year-olds, with nearly equal sex distribution (50.1% male, n=770). Most participants resided in Tula (51.4%, n=791), and the majority reported lifelong residence in their current community (71.2%, n=1,095). Regarding healthcare access, only 15% (n=231) reported regular dental visits, while most had some form of health insurance coverage (65.8%, n=1,012). Maternal literacy rates were high (94.8%, n=1,458).

**Table 1:** Description of the characteristics of the schoolchildren included in the study Data is presented as numbers and %. SEP: socioeconomic position

Variable	Frequency	Percentage
Age		
12 years	688	44.7
15 years	850	55.3
Sex		
Boys	770	50.1
Girls	768	49.9
Current residence		
Tula	791	51.4
El Llano	175	11.4
San Marcos	572	37.2
Lifelong residence		
Yes	1,095	71.2
No	443	28.8
Regular dental visits		
Yes	231	15.0
No	1,307	85.0
Health insurance		
Uninsured	526	34.2
Insured	1,012	65.8
Maternal literacy		
No	80	5.2
Yes	1,458	94.8
SEP (education)		
1st tertile (lowest)	622	40.4
2nd tertile	516	33.6
3rd tertile (highest)	400	26.0
SEP (occupation)		
1st tertile (lowest)	538	35.0
2nd tertile	521	33.9
3rd tertile (highest)	479	31.1

Socioeconomic position analysis revealed that 40.4% (n=622) of participants fell into the lowest education-based tertile, while 26% (n=400) were in the highest tertile. The occupation-based tertile distribution showed similar patterns (n=538, 35% lowest, n=479, 31.1% highest). The overall prevalence of pit and fissure sealants was low at 1.3% (n=20) (Table 1).

Significant associations with PFS prevalence

Table 2 demonstrates the relationships between PFS presence and various independent variables. While no significant associations were found with age, sex, residence location, health insurance status, or maternal literacy (all p-values > 0.05), several noteworthy patterns emerged.

**Table 2:** Association between PFS presence and sociodemographic and socioeconomic variables Data is presented as numbers and %. *p < 0.05, †p < 0.01, ‡non-parametric test for trend. PFS: pit and fissure sealant, SEP: socioeconomic position

Variable	Absent (number (%))	Present (number (%))	Statistical test	p-value
Age				
12 years	681 (99.0)	7 (1.0)		
15 years	837 (98.5)	13 (1.5)	χ²=0.7765	0.378
Sex				
Boys	763 (99.1)	7 (0.9)		
Girls	755 (98.3)	13 (1.7)	χ²=1.8396	0.175
Current residence				
Tula	777 (98.2)	14 (1.8)		
El Llano	174 (99.4)	1 (0.6)		
San Marcos	567 (99.1)	5 (0.9)	Fisher's exact	0.352
Lifelong residence				
Yes	1,085 (99.1)	10 (0.9)		
No	433 (97.7)	10 (2.3)	χ²=4.4395	0.035*
Regular dental visits				
Yes	222 (96.1)	9 (3.9)		
No	1,295 (99.2)	11 (0.8)	Fisher's exact	0.001†
Health insurance				
Uninsured	518 (98.5)	8 (1.5)		
Insured	1,000 (98.8)	12 (1.2)	χ²=0.3029	0.582
Maternal literacy				
No	80 (100)	0 (0.0)		
Yes	1,438 (98.6)	20 (1.4)	Fisher's exact	0.341
SEP (education)				
1st tertile (lowest)	617 (99.2)	5 (0.8)		
2nd tertile	511 (99.0)	5 (1.0)	χ²=6.1212	0.047*
3rd tertile (highest)	390 (97.5)	10 (2.5)	Test for trend‡	0.027*
SEP (occupation)				
1st tertile (lowest)	535 (99.4)	3 (0.6)		
2nd tertile	516 (99.0)	5 (1.0)	χ²=8.2013	0.017*
3rd tertile (highest)	467 (97.5)	12 (2.5)	Test for trend‡	0.007†

Residential mobility showed a significant association, with adolescents who had changed communities demonstrating higher PFS prevalence (2.3%, n=10) compared to stable residents (0.9%, n=10; p=0.035). Regular dental attendance was strongly associated with PFS presence, showing nearly five times greater prevalence among those with routine dental visits (3.9%, n=9 versus 0.8%, n=11; p=0.001).

**Figure 2:**
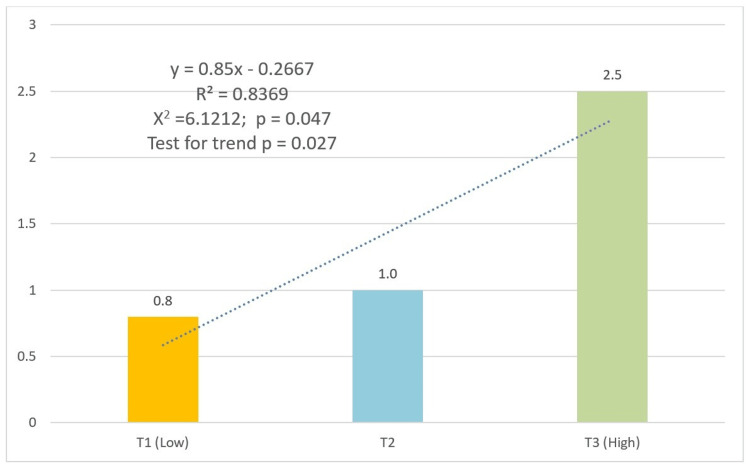
Prevalence of pit and fissure sealants by socioeconomic position tertiles (education) Numbers above bars represent percentages. All group comparisons showed statistically significant differences (p<0.05). Trend lines, equations, and R-squared values were generated in Excel, using the trend line format.

**Figure 3:**
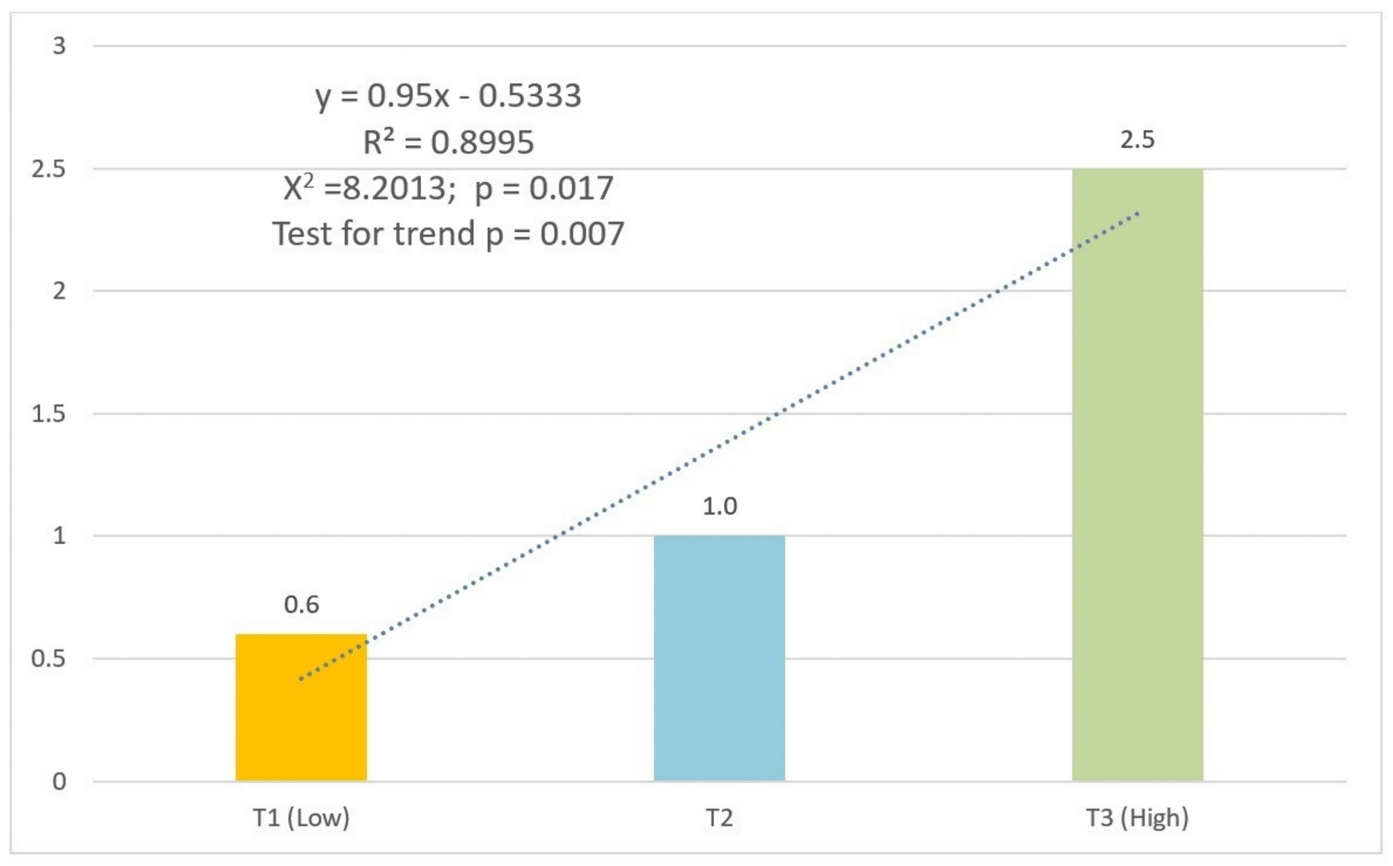
Prevalence of pit and fissure sealants by socioeconomic position tertiles (occupation) Numbers above bars represent percentages. All group comparisons showed statistically significant differences (p<0.05). Trend lines, equations, and R-squared values were generated in Excel, using the trend line format.

Both socioeconomic indicators revealed significant gradients. For education-based SEP, PFS prevalence progressively increased from 0.8% (n=5) in the lowest tertile to 2.5% (n=10) in the highest (χ² p=0.047, test for trend p=0.027) (Figure 2). The occupation-based SEP showed a parallel pattern, with prevalence rising from 0.6% (n=3) to 2.5% (n=12) across tertiles (χ² p=0.017, test for trend p=0.007) (Figure 3).

## Discussion

This cross-sectional study examined the prevalence of pit and fissure sealants (PFS) and their association with socioeconomic factors among Mexican adolescents aged 12 and 15 years. Our findings revealed an exceptionally low PFS prevalence of 1.3% in this population, a rate substantially lower than reported in other settings. Comparative data show markedly higher prevalence in the United States (20%-46%) [19,25,26], Spain (32.2%-34.8%) [27], and even in resource-limited regions such as São Tomé (2.21%) [29], Tibet (3.77%) [30], and Puerto Rico [31]. Previous Mexican studies similarly reported PFS prevalence never exceeding 2.5% over a 10-year period [32].

These striking disparities between Mexico (a developing nation) and high-income countries (United States [19,25,26], Spain [27], and Saudi Arabia [28]) reflect profound oral health inequities and Mexico's persistent curative-oriented dental care model. Multiple systemic factors likely contribute to this gap: absence of institutional preventive programs, budgetary constraints, and socioeconomic barriers. While high-coverage nations implement public health strategies such as school-based PFS applications with subsidies, Mexico's oral health subsystem concentrates sealant services in the private sector, effectively excluding vulnerable populations.

This inequality not only perpetuates Mexico's high childhood caries rates but underscores the urgent need for policy reorientation toward prevention. Cost-effective interventions such as PFS, with demonstrated efficacy in comparable settings [19-23], should be prioritized. The challenges are compounded in low- and middle-income countries by additional barriers including dental workforce shortages, inadequate infrastructure/supplies, and cost-related access limitations [43].

Socioeconomic inequalities and access to preventive services

The study findings confirmed our hypothesis regarding socioeconomic disparities in PFS utilization among Mexican adolescents. We observed significantly higher sealant prevalence in the highest socioeconomic position (SEP) tertile compared to lower tertiles, consistent across both education- and occupation-based indicators. This graded relationship aligns with global evidence linking lower SEP, including subjective income, parental employment status, education level, and access to basic resources, with reduced preventive care access [5,6,39,40].

SEP represents a complex, multidimensional construct in health research. While intuitively understood, its measurement varies considerably, reflecting differing historical and disciplinary perspectives. SEP encompasses the social and economic factors determining an individual's societal position, influencing health through diverse exposures, resources, and vulnerabilities. Importantly, no single optimal SEP indicator exists: different measures capture distinct aspects of socioeconomic stratification, with variable relevance across health outcomes and life stages [39,40,44,45]. In oral health, SEP fundamentally shapes families' capacity to prioritize prevention, mediated through financial constraints, health literacy gaps, and dental service accessibility.

Two additional determinants emerged strongly: residential mobility and dental visit frequency. Adolescents who had relocated showed 2.5 times higher PFS prevalence, potentially reflecting urban service access or selective migration patterns. Regular dental attendees demonstrated nearly fivefold greater sealant rates, underscoring care continuity's importance. However, only 15% of participants reported preventive visits, highlighting Mexico's persistent treatment-oriented dental culture rather than prevention-focused care.

Limitations and strengths

This study has several limitations that should be acknowledged. The cross-sectional design precludes causal inference, and the low prevalence of PFS prevented multivariate analysis. Nevertheless, our work provides two key contributions: (1) representative data from an understudied Mexican population and (2) empirical evidence to inform public health policy decisions.

Future research should prioritize evaluating pilot interventions that combine PFS application with fluoride treatments and oral health education programs, with rigorous measurement of caries incidence reduction. Such implementation studies could establish effective protocols for school-based preventive care in resource-limited settings.

Due to the low frequency of sealant use (only 20 cases in the sample), it was not possible to develop multivariate regression models that controlled for potential confounders. However, the consistent patterns found in the stratified analyses (with p-values < 0.05) support the robustness of the associations identified between socioeconomic status and sealant use.

Implications for public policy

The study's findings provide compelling evidence to guide preventive-focused public health policies, particularly targeting vulnerable populations, in alignment with global health objectives. The stark socioeconomic disparities in PFS prevalence reveal a fragmented oral health system perpetuating inequities, demanding immediate structural interventions through three key pillars.

Universal Prevention Programs With Community-Based Approaches

Systematic school-based PFS campaigns in public schools, especially rural and marginalized areas with limited dental access, should be prioritized. These initiatives must integrate with public social security systems, adapting internationally validated cost-effective models combining PFS with oral health education [19,46]. Evidence shows such programs can reduce permanent molar caries incidence by up to 76% [22], generating a significant return on investment by avoiding complex future treatments.

Primary Care Integration of Oral Health Services

Linking preventive dental services with school health programs and primary care networks (per WHO recommendations [1]) is crucial to overcome access barriers. This requires certain actions such as training general practitioners and nurses in early caries risk identification; including PFS in public health insurance packages; following successful models such as South Korea, where this strategy reduced inequalities in six- to 11-year-olds [47]; and establishing quantifiable coverage targets monitored through health information systems.

Culturally Adapted Community Education

The low preventive care adherence (only 15% regular dental visits) necessitates educational campaigns addressing sociocultural barriers through family/teacher workshops in local languages emphasizing PFS benefits, community leader engagement and local media partnerships to shift oral health perceptions, and government-community partnerships across educational and social sectors, as proposed for Mexico [48].

These measures would not only improve adolescent quality of life but also reduce the economic burden of untreated caries [11], advancing more equitable and sustainable health systems.

## Conclusions

This study clearly shows that very few Mexican teenagers (1.3%, n=20) use pit and fissure sealants (PFS), and it also highlights significant differences in usage based on socioeconomic status. The data show a clear trend where the use of PFS is much higher among teenagers from wealthier backgrounds compared to those from poorer backgrounds, and this pattern is seen in both education and job-related measures of socioeconomic status. Furthermore, the analysis identified a strong positive association between regular dental visits and PFS prevalence. These findings show that factors related to a person's economic and social situation play a key role in how easily they can access this proven method for preventing cavities. Consequently, our results underscore the urgent need for structural reforms prioritizing preventive care and health equity in oral health policy. Achieving this transformation will require coordinated intersectoral collaboration among government entities, academic institutions, and private sector stakeholders to (1) implement empirically validated interventions, (2) overcome the prevailing curative-care paradigm, and (3) ensure equitable dental service access for vulnerable populations.
